# Supplementary material for: A rare variant of African ancestry activates 8q24 lncRNA hub by modulating cancer associated enhancer
Source: Nat Commun. 2020 Jul 17;11:3598. doi: 10.1038/s41467-020-17325-y (PMC7368061; doi:10.1038/s41467-020-17325-y)
Supplement: Supplementary file 1 — Supplementary Information [file 41467_2020_17325_MOESM1_ESM.pdf]

Supplementary Figures and Tables

A rare variant of African ancestry activates 8q24 lncRNA hub by modulating cancer associated enhancer

Walavalkar et al.

A

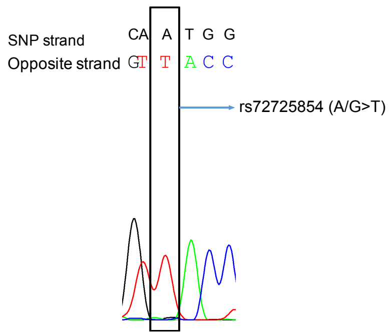

B

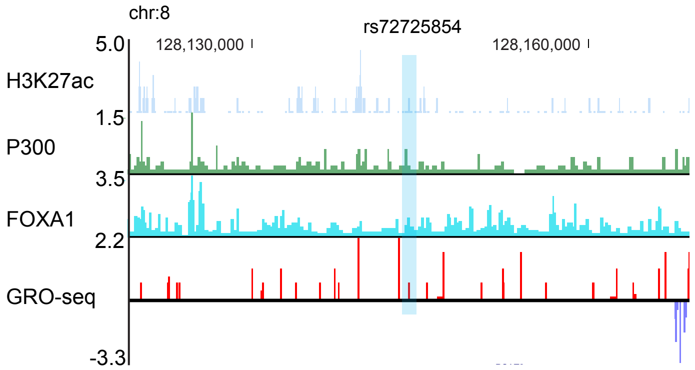

Supplimentary Figure 1. rs72725854 lies in a prostate cancer-specific enhancer:
(A) Sanger sequencing chromatogram shows that LNCaP cell line is homozygous for the non-risk 'A/A' allele of rs72725854. (B) UCSC genome browser shows the H3K27ac, P300, FOXA1 and GRO-seq signal around rs72725854 region in the MCF7 cell line.

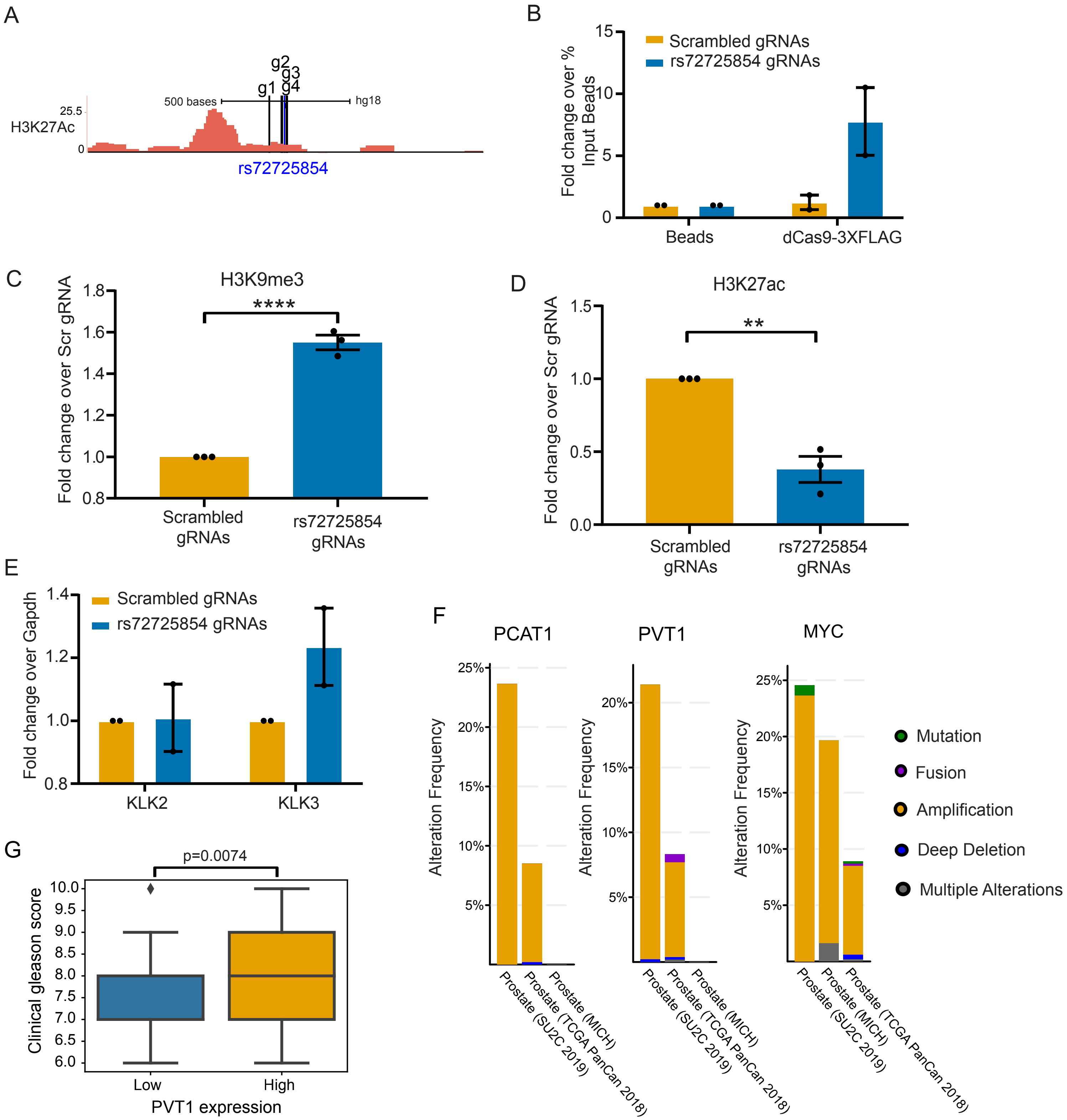

Supplementary Figure 2. rs72725854 is transcriptionally linked with high PCAT1, PVT1 and MYC levels: (A) The enhancer region marked with the location of gRNAs designed to block the enhancer by CRISPRi. (B) ChIP qPCRs depict dCas9-3XFLAG enrichment at the rs72725854 enhancer upon CRISPRi by scr or rs72725854 targeting gRNAs. (C) H3K9me3 and (D) H3K27ac enrichment qPCRs at rs72725854 enhancer upon CRISPRi by scr or rs72725854 targeting gRNAs in LNCaP cells. (E) Expression of KLK2 and KLK3 mRNAs upon CRISPRi by scr or rs72725854 targeting gRNAs in LNCaP cells. Error bars denote SEM from two biological replicates in (B) and (E) and three biological replicates in (C) and (D). (F) Graphs depicting types of alteration in PCAT1, PVT1 and MYC genes in prostate cancers from 3 different datasets. (G) The Gleason score of prostate adenocarcinoma patients with higher vs. lower expression of PVT1 using TCGA dataset. All the patients were divided into 4 quartiles based on PVT1 expression, and patients belonging to q4 are referred here as High PVT1 expression (42 samples) and rest are referred as Low PVT1 expression (237 samples). The boxplot represents minima (Q1-1.5*IQR), first quartile, median, third quartile and maxima (Q3+1.5*IQR). p-values were calculated by Student's two-tailed unpaired t test in (C), (D) and (G). **** p<0.0001, ** p<0.01. Source data are provided as a Source Data file.

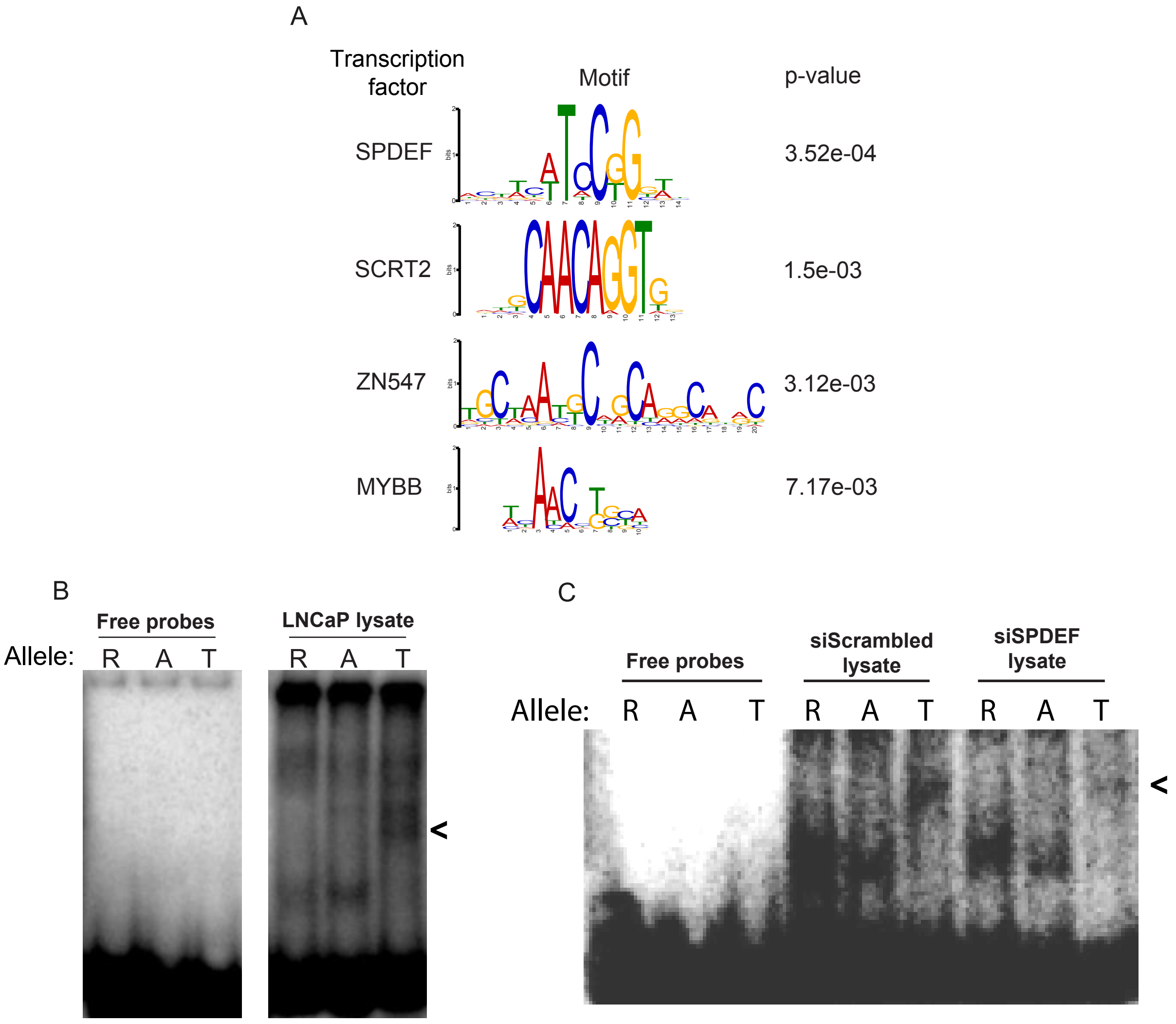

Supplementary Figure 3. SPDEF binds to risk allele 'T': (A) Tomtom motif analysis on rs72725854 region showing the top hits with their respective p-values. The p-values were obtained using Tomtom. (B) EMSA showing the binding of specific proteins on random, A and T oligos using LNCaP nuclear lysate. (C) EMSA showing the protein binding on random, A and T oligos using the nuclear lysate from scr or SPDEF knockdown cells. The experiments in (B) and (C) were performed thrice each. Source data are provided as a Source Data file.

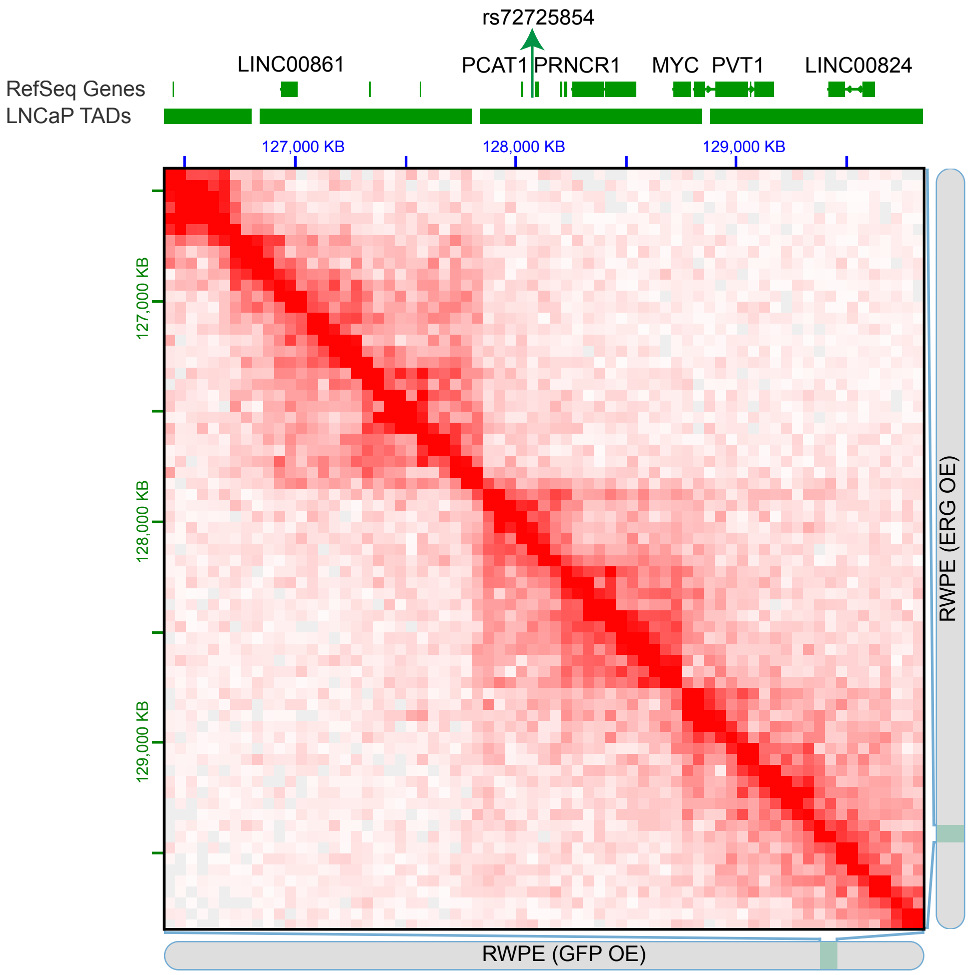

Supplimentary Figure 4. HiC at the 8q24 region in GFP and ERG overexpressing RWPE cells: Differential HiC matrices from GFP and ERG overexpressing RWPE cells are plotted on 8q24 region. The plot is overlaid with gene annotations and TAD locations.

Supplementary Table 1: Sequences of oligos used in the study

**SPDEF-GST bacterial expression**	
SPDEF-GST Forward	GGGGAATTCTCAGATGGGGTGCACGAACTGGTAGACG
SPDEF-GST Reverse	AAAGGATCCGGCAGCGCCAGCCCGGGTCTGAGCA
**SPDEF eukaryotic expression**	
SPDEF Forward	GGGGGATCCTCAGATGGGGTGCACGAACTGGTAGACG
SPDEF Reverse	AAAGAATTCAATGGGCAGCGCCAGCCCGGGTCTGA
**EMSA oligos**	
EMSA-A Forward	TATTTGTTTTGGGTGACACAATTCAACCGGTAACATATCCTGATCCTTGGC
EMSA-A Reverse	GCCAAGGATCAGGATATGTTACCGGTTGAATTGTGTCACCCAAAACAAATA
EMSA-T Forward	TATTTGTTTTGGGTGACACAATTCATCCGGTAACATATCCTGATCCTTGGC
EMSA-T Reverse	GCCAAGGATCAGGATATGTTACCGGATGAATTGTGTCACCCAAAACAAATA
EMSA-Scrambled Forward	GGGGGATCCTCAGATGGGGTGCACGAACTGGTAGACG
EMSA-Scrambled Reverse	AAAGAATTCAATGGGCAGCGCCAGCCCGGGTCTGA
**Luciferase assay oligos**	
rs72725854 Forward	GGTGGTACCGGTCCACCCTAATAATTTCATT
rs72725854 Reverse	GGCCTCGAGGTAGAATCTTCAGTTTACTAAGTTC
rs72725854 A	GGGTGACACAATTCA**A**CCGGTAACATATCC
rs72725854 G	GGGTGACACAATTCA**G**CCGGTAACATATCC
rs72725854 T	GGGTGACACAATTCA**T**CCGGTAACATATCC
**ChIP-qPCR oligos on the enhancer in the plasmid**	
ChIP-qPCR Forward	GGCTCCCTCATCTGCAAAACAAG
ChIP-qPCR Reverse	CCCTCTAGTGTCTAAGCTTGGCC
**qPCR oligos at the enhancer, PCAT1, PRNCR1 and PVT1**	
Pre-PCAT1 Forward	GAGATGACGCAAAGGAACCTAAC
Pre-PCAT1 Reverse	CACTACTCCATGCTGCATCAAC
Pre-PCAT2 Forward	GGTGTCTGACGAAATGATCTGA
Pre-PCAT2 Reverse	CAGAAGGTCTGGTAATAGATGGC
Pre-PRNCR1 Forward	TCAATGGCCCACAAATGTCTAC
Pre-PRNCR1 Reverse	GACCCTCATTTTCCTGCTTCTC
Pre-PVT1 Forward	GTGAGTTACACTCTGTTAGGGCC

Pre-PVT1 Reverse	CTTGAACGAAGCTCCATGCAGC
Pre-c-Myc Forward	CTTCTCTCCGTCCTCGGATTC
Pre-c-Myc Reverse	CCTTCCTAATAAGAGTGGCCCG
eRNA Forward	GGTCACTGGACTGAAGCAGGAAAC
eRNA Reverse	ATCAGGAATAGACCCTTCCCTCACG
KLK2 Forward	CAGGAGTCTTCAGTGTGTGAGC
KLK2 Reverse	GATGGTGTCCTTGATCCACTTCC
KLK3 Forward	GTGTGTGGACCTCCATGTTATTTCC
KLK3 Reverse	CCTTGATCCACTTCCGGTAATGC

ChIP-qpcr oligos on the genomic enhancer

ChIP-qPCR Forward	CGTCTTGTAGTTCTGGAGGCC
ChIP-qPCR Reverse	GAGGGAAGATGGTGTGAGGAG
PSApromoter Forward	CCTACTCTGGAGGAACATATTGTATC
PSApromoter Reverse	GTGTGTCTTCTGAGCAAAGAC
PCAT1intron Forward	GCTCTAACTAAATTCAGTACCCTAGG
PCAT1intron Reverse	CTTTGAATAGGCTCCAAGAGAGG
Negative cntrl 1 Forward	CACCCTTTCAGGCAACCACA
Negative cntrl 1 Reverse	GTCTATAAATTAGCCAAAGGGCTCAG
Negative cntrl 2 Forward	CTGATCCCAAGCCACAAATCC
Negative cntrl 2 Reverse	GGTCATCTTCACATTAACAATGGC

4C oligos for enhancer, PCAT1 and PVT1 viewpoints

Enhancer Forward	AATGATACGGCGACCACCGAACACTCTTTCCCTACACGACGCTCTTCCGATCTGTAATGAGAGGCCCCAAAGCTT
Enhancer Reverse	CAAGCAGAAGACGGCATACGAAGAACTGTTACTATGCATCATGTAGATGC
PCAT1 Forward	AATGATACGGCGACCACCGAACACTCTTTCCCTACACGACGCTCTTCCGATCTTGCCAAGGACACGTAGAAGCTT
PCAT1 Reverse	CAAGCAGAAGACGGCATACGAGATAGCCAATGGAGATGATTTAATGGGG
PVT1 Forward	AATGATACGGCGACCACCGAACACTCTTTCCCTACACGACGCTCTTCCGATCTGTATTGAAGAGATGGAAGCTT
PVT1 Reverse	CAAGCAGAAGACGGCATACGAACAGGAAGCCAAGACTAGTT

gRNAs for CRISPRi and CRISPR deletion

rs72725854(g1)	ggagaaCCACCTTGTTGGCCCCAGAGTGTGACCGTATTGTTTTAGAGCTAGAAATAGCAAGTT
rs72725854(g2)	ggagaaCCACCTTGTTGGTTTGGGTGACACAATTCAACGTTTTAGAGCTAGAAATAGCAAGTT
rs72725854(g3)	ggagaaCCACCTTGTTGGCAAGGATCAGGATATGTTACGTTTTAGAGCTAGAAATAGCAAGTT
rs72725854(g4)	ggagaaCCACCTTGTTGGCGGTAACATATCCTGATCCTGTTTTAGAGCTAGAAATAGCAAGTT
gRNA Reverse	AAACCCAAGGATCAGGATATGTTACCGC
gRNA sequencing oligo	CACCGCGGTAACATATCCTGATCCTTGG

DNase-qPCR

ChIP Enhancer Forward	CGTCTTGTAGTTCTGGAGGCC
ChIPEnhancer Reverse	GAGGGAAGATGGTGTGAGGAG
Amp Forward	CGGTCAAGCCGGGTCACATG
Amp Reverse	GAGCGACAACACCGCGGCCAAC

Sppelementary Table 2: datasets used in the study:

GEO accession numbers	Data
GSM1543778	LNCaP GRO-seq
GSM1543782	LNCaP GRO-seq
GSM1543788	LNCaP GRO-seq
GSM1115995	MCF7 GRO-seq
GSM1115996	MCF7 GRO-seq
GSM1716762	FOXA1 tumors
GSM686925	FOXA1 LNCaP
ENCODE	DHS LNCaP
ENCODE	DHS PrEC
GSM1862989	H3K27ac ChIP-seq LNCaP
GSM1383865	H3K27ac PrEC
ENCODE	DHS-seq cell lines
SRX021799	AR ChIP-seq VCaP
SRR054898	SPDEF ChIP-seq VCaP
GSM2827606	H3K27ac ChIP-seq VCaP
GSM1328964	PolII ChIP-seq VCaP
GSM3567207	Med1 ChIP-seq VCaP
GSM2537225	FoxA1 ChIP-seq VCaP
GSM927075/77/78/79/80	Hi-C RWPE with ERG overexpression
GSM927076/81/82/83/84	Hi-C RWPE with GFP overexpression
GSM2827298/99	Hi-C LNCaP
GSM2827515/16	Hi-C T47D

Supplemetary Table 3: Number of 4C reads

4C viewpoint	No. of reads (million)
Enhancer replicate 1	3.96
PCAT1 replicate 1	18.56
PVT1 replicate 1	30.27
Enhancer replicate 2	4.54
PCAT1 replicate 2	6.13
PVT1 replicate 2	8.99
