# Supplementary material for: A rare variant of African ancestry activates 8q24 lncRNA hub by modulating cancer associated enhancer
Source: Nat Commun. 2020 Jul 17;11:3598. doi: 10.1038/s41467-020-17325-y (PMC7368061; doi:10.1038/s41467-020-17325-y)
Supplement: Supplementary file 3 — Reporting Summary [file 41467_2020_17325_MOESM3_ESM.pdf]

nature research

Corresponding author(s): Dimple Notani

Last updated by author(s): 2020/03/18

Reporting Summary

Nature Research wishes to improve the reproducibility of the work that we publish. This form provides structure for consistency and transparency in reporting. For further information on Nature Research policies, see Authors & Referees and the Editorial Policy Checklist.

Statistics

For all statistical analyses, confirm that the following items are present in the figure legend, table legend, main text, or Methods section.

n/a	Confirmed	
☐	☒	The exact sample size (n) for each experimental group/condition, given as a discrete number and unit of measurement
☐	☒	A statement on whether measurements were taken from distinct samples or whether the same sample was measured repeatedly
☐	☒	The statistical test(s) used AND whether they are one- or two-sided<br>*Only common tests should be described solely by name; describe more complex techniques in the Methods section.*
☒	☐	A description of all covariates tested
☒	☐	A description of any assumptions or corrections, such as tests of normality and adjustment for multiple comparisons
☐	☒	A full description of the statistical parameters including central tendency (e.g. means) or other basic estimates (e.g. regression coefficient) AND variation (e.g. standard deviation) or associated estimates of uncertainty (e.g. confidence intervals)
☐	☒	For null hypothesis testing, the test statistic (e.g. F, t, r) with confidence intervals, effect sizes, degrees of freedom and P value noted<br>*Give P values as exact values whenever suitable.*
☒	☐	For Bayesian analysis, information on the choice of priors and Markov chain Monte Carlo settings
☒	☐	For hierarchical and complex designs, identification of the appropriate level for tests and full reporting of outcomes
☒	☐	Estimates of effect sizes (e.g. Cohen's d, Pearson's r), indicating how they were calculated

Our web collection on statistics for biologists contains articles on many of the points above.

Software and code

Policy information about availability of computer code

Data collection	No software was used for data collection
Data analysis	ENCODE, SRA, TCGA, ICGC, PCAWG, cbioportal, CCLE portal, GTEX, https://github.com/rr1859/R.4Cker, Bowtie2 V2.3.0 , HOMER V4.10.4, TOMTOM-MEME V5.0.5, Juicebox V1.11.08, Juicer V1.5.6, gprofiler e96_eg43_p13_3a389c1, REVIGO. Plots were made using Prism 6 (GraphPad)

For manuscripts utilizing custom algorithms or software that are central to the research but not yet described in published literature, software must be made available to editors/reviewers. We strongly encourage code deposition in a community repository (e.g. GitHub). See the Nature Research guidelines for submitting code & software for further information.

Data

Policy information about availability of data

All manuscripts must include a data availability statement. This statement should provide the following information, where applicable:

- Accession codes, unique identifiers, or web links for publicly available datasets
- A list of figures that have associated raw data
- A description of any restrictions on data availability

Mutations, gene expression, and clinical annotation of cancer patient samples were obtained from the publicly available repositories: PCAWG (https://dcc.icgc.org/pcawg), TCGA Genomic Data Commons (GDC) (https://portal.gdc.cancer.gov/) and cBioPortal (https://www.cbioportal.org/). The ATAC-seq data of prostate tumors in TCGA was obtained from https://gdc.cancer.gov/about-data/publications/ATACseq-AWG. Normalized RNA-seq data of normal and tumor tissues (in TCGA and GTEx) was obtained from Wang et al., 2018 (53). The cancer cell line data was obtained from CCLE (https://portals.broadinstitute.org/ccle) and ENCODE (https://www.encodeproject.org/). The data in various graphs in main figures (Fig. 2A, 2B, 2D, 2E; Fig. 3B, 3C, 3D; Fig. 4C; Fig. 5C, 5D, 5E, 5F, 5G, 5H, 5I; Fig. 6F), Supplementary figures (2B, 2C, 2D and 2E) and full images of EMSA gel (Fig. 5B; Supplementary Fig. 3B, 3C) immunoblots (Fig. 5D) are provided in source data. The bedGraph data for the 4C plots (Fig. 3) is also provided in the source data.

Field-specific reporting

Please select the one below that is the best fit for your research. If you are not sure, read the appropriate sections before making your selection.

☒ Life sciences ☐ Behavioural & social sciences ☐ Ecological, evolutionary & environmental sciences

For a reference copy of the document with all sections, see nature.com/documents/nr-reporting-summary-flat.pdf

Life sciences study design

All studies must disclose on these points even when the disclosure is negative.

Sample size	No statistical methods were used for sample size. Multiple biological replicates with technical replicates were performed to test the significance. This is the standard norm in the field
Data exclusions	No data were excluded
Replication	Reproducibility of the results were ensured by involving multiple members of the lab to collect data and by repeating experiments multiple times. Some biological replicates on CRISPRi lines were performed after long intervals to assess the reproducibility of the data.
Randomization	Randomization was not performed
Blinding	Two authors independently performed gene expression analysis to ensure the reproducibility. Blinding was done for chromatin feature experiment and for the plasmids with different alleles in reporter assays.

Reporting for specific materials, systems and methods

We require information from authors about some types of materials, experimental systems and methods used in many studies. Here, indicate whether each material, system or method listed is relevant to your study. If you are not sure if a list item applies to your research, read the appropriate section before selecting a response.

Materials & experimental systems

n/a	Involved in the study	
☐	☒	Antibodies
☐	☒	Eukaryotic cell lines
☒	☐	Palaeontology
☒	☐	Animals and other organisms
☒	☐	Human research participants
☒	☐	Clinical data

Methods

n/a	Involved in the study	
☒	☐	ChIP-seq
☒	☐	Flow cytometry
☒	☐	MRI-based neuroimaging

Antibodies

Antibodies used	SPDEF (sc-166846, Santacruz Biotechnology), FLAG (F7425, Sigma), H3K27ac (ab4729, Abcam) and H3K9me3 (39161, Active Motif). GAPDH (sc-32233, Santacruz Biotechnology)
Validation	SPDEF (sc-166846, Santacruz Biotechnology) - (https://www.scbt.com/p/pdef-antibody-g-10)<br>FLAG (F7425, Sigma Aldrich) - (https://www.sigmaaldrich.com/catalog/product/sigma/f7425?lang=en&region=IN)<br>H3K27ac (ab4729, Abcam) - (https://www.abcam.com/histone-h3-acetyl-k27-antibody-chip-grade-ab4729.html)<br>H3K9me3 (39161, Active Motif) - (https://www.activemotif.com/catalog/details/39765/histone-h3-trimethyl-lys9-antibody-pab-1)<br>GAPDH (sc-32233, Santacruz Biotechnology) - (https://www.scbt.com/p/gapdh-antibody-6c5)

Eukaryotic cell lines

Policy information about cell lines

Cell line source(s)	LNCaP (ATCC), 293FT (ATCC)
Authentication	LNCAP cells were authenticated by their response to androgens. The response was monitored by RTPCRs, Chromatin immunoprecipitations and reporter assays. 293FT were authenticated by sanger sequencing.
Mycoplasma contamination	Cell lines were tested and were found to be negative for mycoplasma

Commonly misidentified lines<br>(See ICLAC register)	These cell lines are not misidentified
